# Discovering HIV related information by means of association rules and machine learning

**DOI:** 10.1038/s41598-022-22695-y

**Published:** 2022-10-28

**Authors:** Lourdes Araujo, Juan Martinez-Romo, Otilia Bisbal, Ricardo Sanchez-de-Madariaga, The Cohort of the National AIDS Network (CoRIS), Joaquín Portilla, Irene Portilla, Esperanza Merino, Gema García, Iván Agea, José Sánchez-Payá, Juan Carlos Rodríguez, Livia Giner, Sergio Reus, Vicente Boix, Diego Torrus, Verónica Pérez, Julia Portilla, Juan Luís Gómez, Jehovana Hernández, Ana López Lirola, Dácil García, Felicitas Díaz-Flores, M. Mar Alonso, Ricardo Pelazas, M. Remedios Alemán, Víctor Asensi, María Eugenia Rivas Carmenado, Tomás Suarez-Zarracina, Federico Pulido, Rafael Rubio, Otilia Bisbal, M. Asunción Hernando, David Rial, María de Lagarde, Octavio Arce, Adriana Pinto, Laura Bermejo, Mireia Santacreu, Roser Navarro, Candela Gonzalez, Jose Antonio Iribarren, M. José Aramburu, Xabier Camino, Miguel Ángel von Wichmann, Miguel Ángel Goenaga, M. Jesús Bustinduy, Harkaitz Azkune, Maialen Ibarguren, Xabier Kortajarena, Ignacio Álvarez-Rodriguez, Leire Gil, Lourdes Martínez, Félix Gutiérrez, Catalina Robledano, Mar Masiá, Sergio Padilla, Araceli Adsuar, Rafael Pascual, Marta Fernández, Antonio Galiana, José Alberto García, Xavier Barber, Vanessa Agullo, Javier Garcia Abellán, Reyes Pascual, Guillermo Telenti, Lucia Guillén, Ángela Botella, Roberto Muga, Arantza Sanvisens, Daniel Fuster, Juan Berenguer, Isabel Gutierrez, Juan Carlos López, Margarita Ramírez, Belén Padilla, Paloma Gijón, Teresa Aldamiz-Echevarría, Francisco Tejerina, Cristina Diez, Leire Pérez, Chiara Fanciulli, Saray Corral, Francesc Vidal, Anna Martí, Joaquín Peraire, Consuelo Viladés, Montserrat Vargas, Montserrat Olona, Anna Rull, Verónica Alba, Elena Yeregui, Jenifer Masip, Graciano García-Pardo, Frederic Gómez Bertomeu, Sonia Espineira, Marta Montero, Sandra Cuéllar, Marino Blanes, María Tasias, Eva Calabuig, Miguel Salavert, Juan Fernández, Inmaculada Segarra, Juan González-García, Ana Delgado-Hierro, José Ramón Arribas, Victor Arribas, Jose Ignacio Bernardino, Carmen Busca, Joanna Cano, Julen Cardiñanos, Juan Miguel Castro, Luis Escosa, Iker Falces, Pedro Herranz, Victor Hontañón, Milagros García, Alicia González-Baeza, Ma Luz Martín-Carbonero, Mario Mayoral, Ma Jose Mellado, Rafael Micán, Rosa de Miguel, Rocío Montejano, Ma Luisa Montes, Victoria Moreno, Luis Ramos, Berta Rodés, Talía Sainz, Elena Sendagorta, Eulalia Valencia, Jose Ramón Blanco, Laura Pérez-Martínez, José Antonio Oteo, Valvanera Ibarra, Luis Metola, Mercedes Sanz, Piedad Arazo, Gloria Sampériz, David Dalmau, Marina Martinez, Angels Jaén, Montse Sanmartí, Mireia Cairó, Javier Martinez-Lacasa, Pablo Velli, Roser Font, Mariona Xercavins, Noemí Alonso, Francesco Aiello, María Rivero, Beatriz Piérola, Maider Goikoetxea, María Gracia, Carlos Ibero, Estela Moreno, Jesús Repáraz, Gemma Navarro, Manel Cervantes Garcia, Sonia Calzado Isbert, Marta Navarro Vilasaro, Belen Lopez Garcia, Ignacio de los Santos, Alejandro de los Santos, Jesús Sanz, Lucio García-Fraile, Enrique Martín, Ildefonso Sánchez-Cerrillo, Marta Calvet, Ana Barrios, Azucena Bautista, Carmen Sáez, Marianela Ciudad, Ángela Gutiérrez, Santiago Moreno, Santos del Campo, José Luis Casado, Fernando Dronda, Ana Moreno, M. Jesús Pérez, Sergio Serrano, Ma Jesús Vivancos, Javier Martínez-Sanz, Alejandro Vallejo, Matilde Sanchez, Jose Antonio Pérez-Molina, José Manuel Hermida, Enrique Bernal, Antonia Alcaraz, Joaquín Bravo, Ángeles Muñoz, Cristina Tomás, Mónica Martínez, M. Carmen Villalba, Federico García, Clara Martínez, José Hernández, Leopoldo Muñoz Medina, Marta Álvarez, Natalia Chueca, David Vinuesa, Adolfo de Salazar, Ana Fuentes, Emilio Guirao, Laura Viñuela, Andrés Ruiz-Sancho, Francisco Anguita, Jorge Del Romero, Montserrat Raposo, Carmen Rodríguez, Teresa Puerta, Juan Carlos Carrió, Mar Vera, Juan Ballesteros, Oskar Ayerdi, Begoña Baza, Eva Orviz, Antonio Antela, Elena Losada, Melchor Riera, María Peñaranda, M. Angels Ribas, Antoni A. Campins, Mercedes Garcia-Gazalla, Francisco J. Fanjul, Javier Murillas, Francisco Homar, Helem H. Vilchez, Luisa Martin, Antoni Payeras, Jesús Santos, María López, Crisitina Gómez, Isabel Viciana, Rosario Palacios, Luis Fernando López-Cortés, Nuria Espinosa, Cristina Roca, Silvia Llaves, Juan Manuel Tiraboschi, Arkaitz Imaz, Ana Karina Silva, María Saumoy, Sofía Catalina Scévola, Adrián Curran, Vicenç Falcó, Jordi Navarro, Joaquin Burgos, Paula Suanzes, Jorge García, Vicente Descalzo, Patricia Álvarez, Bibiana Planas, Marta Sanchiz, Lucía Rodríguez, Julián Olalla, M. José Sánchez, Javier Pérez, Alfonso del Arco, Javier de la Torre, José Luis Prada, Onofre Juan Martínez, Lorena Martinez, Francisco Jesús Vera, Josefina García, Begoña Alcaraz, Antonio Jesús Sánchez Guirao, Alvaro Mena, Angeles Castro, Berta Pernas, Pilar Vázquez, Soledad López, Sofía Ibarra, Guillermo García, Josu Mirena, Oscar Luis Ferrero, Josefina López, M. Mar Cámara, Mireia de la Peña, Miriam Lopez, Iñigo Lopez, Itxaso Lombide, Victor Polo, Joana de Miguel, Carlos Galera, Marian Fernández, Helena Albendin, Antonia Castillo, Asunción Iborra, Antonio Moreno, M. Angustias Merlos, Asunción Vidal, Concha Amador, Francisco Pasquau, Concepcion Gil, Jose Tomás Algado, Inés Suarez-García, Eduardo Malmierca, Patricia González-Ruano, M. Pilar Ruiz, José Francisco Pascual, Elena Sáez, Luz Balsalobre, M. Villa López, Mohamed Omar, Carmen Herrero, M. Amparo Gómez, Miguel Alberto de Zarraga, Desiré Pérez, Vicente Estrada, Nieves Sanz, Noemí Cabello, Jorge Vergas García, Maria Jose Núñez, Iñigo Sagastagoitia, Miguel Górgolas, Alfonso Cabello, Beatriz Álvarez, Laura Prieto, Irene Carrillo, José Sanz, Alberto Arranz, Cristina Hernández, María Novella, M. José Galindo, Ana Ferrer, Antonio Rivero Román, Inma Ruíz, Antonio Rivero Juárez, Pedro López, Isabel Machuca, Mario Frias, Ángela Camacho, Ignacio Pérez, Diana Corona, Ignacio Pérez, Diana Corona, Miguel Cervero, Rafael Torres, Juan Antonio Pineda, Pilar Rincón, Juan Macías, Luis Miguel Real, Anais Corma, Marta Fernández, Alejandro Gonzalez-Serna, Eva Poveda, Alexandre Pérez, Luis Morano, Celia Miralles, Antonio Ocampo, Guillermo Pousada, Lucía Patiño, Carlos Dueñas, Sara Gutiérrez, Elena Tapia, Cristina Novoa, Xjoylin Egües, Pablo Telleria

**Affiliations:** 1 Languages and Information Systems Dpt., ETS Ingeniería Informática (UNED), Juan del Rosal 16, 28040 Madrid, Spain; 2 Hospital Universitario 12 de Octubre, Instituto de Investigación I+12, Madrid, Spain; 3 Telemedicine and e-Health Research Unit, Instituto de Salud Carlos III, Monforte de Lemos 5, 28029 Madrid, Spain; 4 Instituto Mixto UNED-ISCIII IMIENS, 28029 Madrid, Spain; 5 Hospital General Universitario de Alicante, Alicante, Spain; 6 Hospital Universitario de Canarias, San Cristóbal de la Laguna, Spain; 7 Hospital Universitario Central de Asturias, Oviedo, Spain; 8 Hospital Universitario 12 de Octubre, Madrid, Spain; 9 Servicio de Enfermedades Infecciosas, Hospital Universitario Donostia, Instituto de Investigación BioDonostia, Donostia-San Sebastián , Spain; 10 Hospital General Universitario De Elche, Elche, Spain; 11 Hospital Universitari Germans Trias i Pujol (Can Ruti), Badalona, Spain; 12 Hospital General Universitario Gregorio Marañón, Madrid, Spain; 13 Hospital Universitari de Tarragona Joan XXIII, Tarragona, Spain; 14 Hospital Universitario y Politécnico de La Fe, Valencia, Spain; 15 Hospital Universitario La Paz/IdiPAZ, Madrid, Spain; 16 Hospital San Pedro Centro de Investigación Biomédica de La Rioja (CIBIR), Logroño, Spain; 17 Hospital Universitario Miguel Servet, Zaragoza, Spain; 18 Hospital Universitari Mutua Terrassa, Terrassa, Spain; 19 Complejo Hospitalario de Navarra, Pamplona, Spain; 20 Parc Taulí Hospital Universitari, Sabadell, Spain; 21 Hospital Universitario de La Princesa, Madrid, Spain; 22 Hospital Universitario Ramón y Cajal, Madrid, Spain; 23 Hospital General Universitario Reina Sofía, Murcia, Spain; 24 Hospital Nuevo San Cecilio, Granada, Spain; 25 Centro Sanitario Sandoval, Madrid, Spain; 26 Hospital Clínico Universitario de Santiago, Santiago de Compostela, Spain; 27 Hospital Universitario Son Espases, Palma de Mallorca, Spain; 28 Hospital Universitario Virgen de la Victoria, Málaga, Spain; 29 Hospital Universitario Virgen del Rocío, Seville, Spain; 30 Hospital Universitario de Bellvitge, Hospitalet de Llobregat, Spain; 31 Hospital Universitario Valle de Hebrón, Barcelona, Spain; 32 Hospital Costa del Sol, Marbella , Spain; 33 Hospital General Universitario Santa Lucía, Cartagena, Spain; 34 Complejo Hospitalario Universitario a Coruña (Chuac), A Coruña, Spain; 35 Hospital Universitario Basurto, Bilbao, Spain; 36 Hospital Universitario Virgen de la Arrixaca, El Palmar, Spain; 37 Hospital de la Marina Baixa, La Vila Joiosa, Spain; 38 Hospital Universitario Infanta Sofía, San Sebastián de los Reyes, Spain; 39 Hospital Universitario de Jaén, Jaén, Spain; 40 Hospital Universitario San Agustín, Avilés, Spain; 41 Hospital Clínico San Carlos, Madrid, Spain; 42 Hospital Universitario Fundación Jiménez Díaz, Madrid, Spain; 43 Hospital Universitario Príncipe de Asturias, Alcalá de Henares, Spain; 44 Hospital Clínico Universitario de Valencia, Valencia, Spain; 45 Hospital Reina Sofía, Córdoba, Spain; 46 Hospital Universitario Severo Ochoa, Leganés, Spain; 47 Nuestra Señora de Valme, Seville, Spain; 48 Hospital Álvaro Cunqueiro, Vigo, Spain; 49 Hospital Clínico Universitario de Valladolid, Valladolid, Spain

**Keywords:** HIV infections, Computer science, Information technology

## Abstract

Acquired immunodeficiency syndrome (AIDS) is still one of the main health problems worldwide. It is therefore essential to keep making progress in improving the prognosis and quality of life of affected patients. One way to advance along this pathway is to uncover connections between other disorders associated with HIV/AIDS—so that they can be anticipated and possibly mitigated. We propose to achieve this by using Association Rules (ARs). They allow us to represent the dependencies between a number of diseases and other specific diseases. However, classical techniques systematically generate every AR meeting some minimal conditions on data frequency, hence generating a vast amount of uninteresting ARs, which need to be filtered out. The lack of manually annotated ARs has favored unsupervised filtering, even though they produce limited results. In this paper, we propose a semi-supervised system, able to identify relevant ARs among HIV-related diseases with a minimal amount of annotated training data. Our system has been able to extract a good number of relationships between HIV-related diseases that have been previously detected in the literature but are scattered and are often little known. Furthermore, a number of plausible new relationships have shown up which deserve further investigation by qualified medical experts.

## Introduction

According to information provided by World Health Organization (WHO), HIV/AIDS remains one of the world’s most serious public health problems, particularly in low and middle-income countries. The development of AIDS (acquired immunodeficiency syndrome) disease, in patients infected with HIV, causes a progressive deterioration of the immune system and decreases the person’s ability to fight many infections and other diseases as well. AIDS refers to the most advanced stages of HIV infection and is defined by the development of one or more opportunistic infections or related cancers among many other possibilities.

WHO has released a number of policy guidelines to assist countries in implementing programs to improve HIV prevention, treatment, care and support services for affected patients. Several initiatives are underway in each country along these lines. One of these initiatives in Spain has been the launching of the Spanish HIV/AIDS Research Network whose main goal consists of improving the health and quality of those affected. This network has generated the HIV/AIDS Research Network Cohort (CoRIS), which makes available to researchers the data from its main database and associated satellite databases, linking biological samples. CoRIS is an open, prospective and multicenter cohort of adult subjects with confirmed HIV infection, launched in 2004. Patients over 13 years old, and naive to antirretroviral treatment (ART) at study entry, have been recruited in HIV care units of the Spanish Public Health System and all of them have signed an informed consent form.

HIV is associated with the development of a large number of other diseases. In some cases, these diseases can be more or less mild and transient. However, in other cases, they can be very serious and long-lasting. It is essential to know the possible relationships between this diversity of diseases associated with HIV, since increasing the knowledge about them can be of great help in their diagnosis and prevention, thus improving the patients’ quality of life. Knowledge about the conditions that typically appear with these diseases, and in what form, will help in making decisions about their prevention and treatment.

In this study, we have focused on applying machine learning (ML) techniques to extract information about the relationships between diseases associated with HIV. Specifically, we have turned to the extraction of association rules of high reliability and coverage to identify relevant relationships between HIV-related diseases.

Association rules (ARs)^1^ are a data mining method that aims to discover patterns of co-occurrence between items in a transactional database. Specifically, we consider a set of *n* items $I= \{i_1,i_2,\ldots ,i_n\}$ and a set or database of transactions on these items: $I= \{i_1,i_2,\ldots ,i_n\}$. Each transaction is represented by a subset of items ($I= \{i_1,i_2,\ldots ,i_d\}$) that have occurred simultaneously.

ARs present the following form:

$$\begin{aligned} X \Rightarrow Y \end{aligned}$$

where *X* and *Y* are two disjoint sets of items. We focus on a particular type of rules whose consequent is a single element.

An example of an association rule, that we could find in a database in which the transactions are the number of diseases suffered by the same patient, could be the following:

*Urinary tract infection, abdominal pain, diabetes* $\Rightarrow$ *renal failure*

ARs have been frequently applied to the medical domain for different purposes. Imamura et al.^2^ applied them to find clinical findings associated with diseases. They have also been used to analyze patterns of lifestyle risk behaviors including smoking, heavy drinking, physical inactivity^3^. There have also been proposals that applied ARs to find relationships between healthcare parameters and specific diseases^4^, such as antimicrobial resistance^5^, psoriasis^6^, COVID-19^7^, or Hospital-Acquired Infections^8^, among others.

Several algorithms have been proposed to systematically generate all ARs that satisfy certain favorable conditions. These conditions refer to parameters such as support (how frequent an itemset is in the transaction set) and confidence (the likeliness of occurrence of consequents in the set, given that the set already has the antecedents).

One of the algorithms that allow the generation of association rules from frequent itemsets is the Frequent-Pattern Growth or FP-Growth algorithm^9^.

By applying this or similar algorithms, we can generate the whole set of rules that satisfy the specified minimum support and minimum confidence thresholds. However, this process leads to a huge number of rules, many of which are uninteresting. Actually, discovering interesting or relevant rules is a difficult problem^10–12^ that needs to be tackled. For example, considering the following AR:

*pollen allergy* $\Rightarrow$ *renal failure*

Since pollen allergy is a very common problem, it can appear in many rules as an antecedent or a consequent. Therefore, ARs, such as the one appearing above, may meet the required frequency threshold, and yet not provide relevant information.

A relevant rule is one that includes at least one relevant relationship between some disease of the antecedent and the one of the consequent. Relevant relationships are those validated by medical experts.

Filtering relevant or interesting rules is a difficult problem that is primarily tackled using unsupervised approaches that do not require expert-annotated training data. These methods attempt to find hidden patterns from raw unlabeled data. Among these unsupervised approaches are those based on associating to the AR a p-value (the likelihood that the association is spurious due to chance)^13,14^. Specifically, the p-value of an AR R is the probability of observing R, or one rule stricter than R, when the two sides of R are independent. If a rule found in the data has a low p-value, it is unlikely that the two sides are independent. Rules with high p-values do not provide information about the independence of the two sides of the rule and can be discarded, as they have most likely appeared by pure chance.

However, unsupervised methods have a limit to the accuracy they can achieve. These limitations can be addressed by using supervised methods that are capable of learning from specific relationship examples, taking into account aspects beyond frequencies.

Supervised methods require labeled data that are used to extract knowledge. These methods start by applying a training process with a labeled data set and try to infer a function that fits the training data appropriately. Then, when applied on new data, this function is able to predict the output. In the case of ARs, few supervised systems have been proposed due to the lack of labeled data.

One way to circumvent this problem is to use the semi-supervised^15^ approach, which employs both labeled and unlabeled data in the training process. This approach typically uses a small amount of labeled data and a larger amount of raw data. Techniques based on this approach can be adjusted to improve their performance as they have larger amounts of training data, for example in a feedback process.

Sánchez-de-Madariaga et al.^16^ proposed a new semi-supervised data mining model, EXTRAE, that combines unsupervised techniques (p-value computed as Fisher’s exact test) with highly limited supervision. The training process starts with a small seed of annotated data, and the model improves its results (F-measure) using a fully supervised system (standard supervised machine learning algorithms). The key idea of this proposal is to enlarge the size of the training data by checking the agreement between the predictions of the supervised system and those of the unsupervised techniques in a series of iterative steps. The remarkable feature of this system is its ability to improve the results of purely supervised methods by combining them with unsupervised techniques. This system has been evaluated on data from the medical domain. Specifically, it has been evaluated on a set of ARs generated from primary care data, which have been manually labeled as true or false (of no interest). The diseases considered were rather common as they came from primary care data and the association rules generated are of limited interest. However, the results obtained showed the potential of the method designed.

The main goal of this study is to find poorly understood relationships between HIV-associated diseases using association rules. To this end, we propose to apply and adapt the semi-supervised algorithm with minimal supervision^16^ mentioned above, to filter association rules between HIV-related diseases. To apply the algorithm and also to be able to perform a proper evaluation, we have started by generating a dataset of association rules and then manually labeling a part of them as relevant or not by expert doctors in HIV. The rules considered by the doctor come from applying the FP-Growth algorithm to the CoRIS cohort data. The input data to our algorithm are ARs annotated with a label indicating whether they are relevant or not.

Since, to the best of our knowledge, there is little history of annotation of ARs as relevant or not, this annotation process has required the definition of a new annotation guideline, this being a contribution of the present study. Another important contribution is the associations found between diseases themselves. We also provide a highly accurate method for the classification of ARs as relevant or not, which has been evaluated on the previously created dataset.

In summary, the main contributions of the present study are outlined as follows:

- An analysis of the most appropriate conditions for assigning the relevance of the rules based on the relationships between the diseases contained in them.
- A collection of association rules between HIV-associated diseases labeled as relevant or not by experts.
- Design of a semi-supervised system that requires only a very small amount of annotated data and is able to predict with high accuracy whether new ARs for HIV-associated diseases are relevant or not.
- An expert analysis of various relationships between HIV-related diseases revealed by the ARs found.

**Figure 1:**
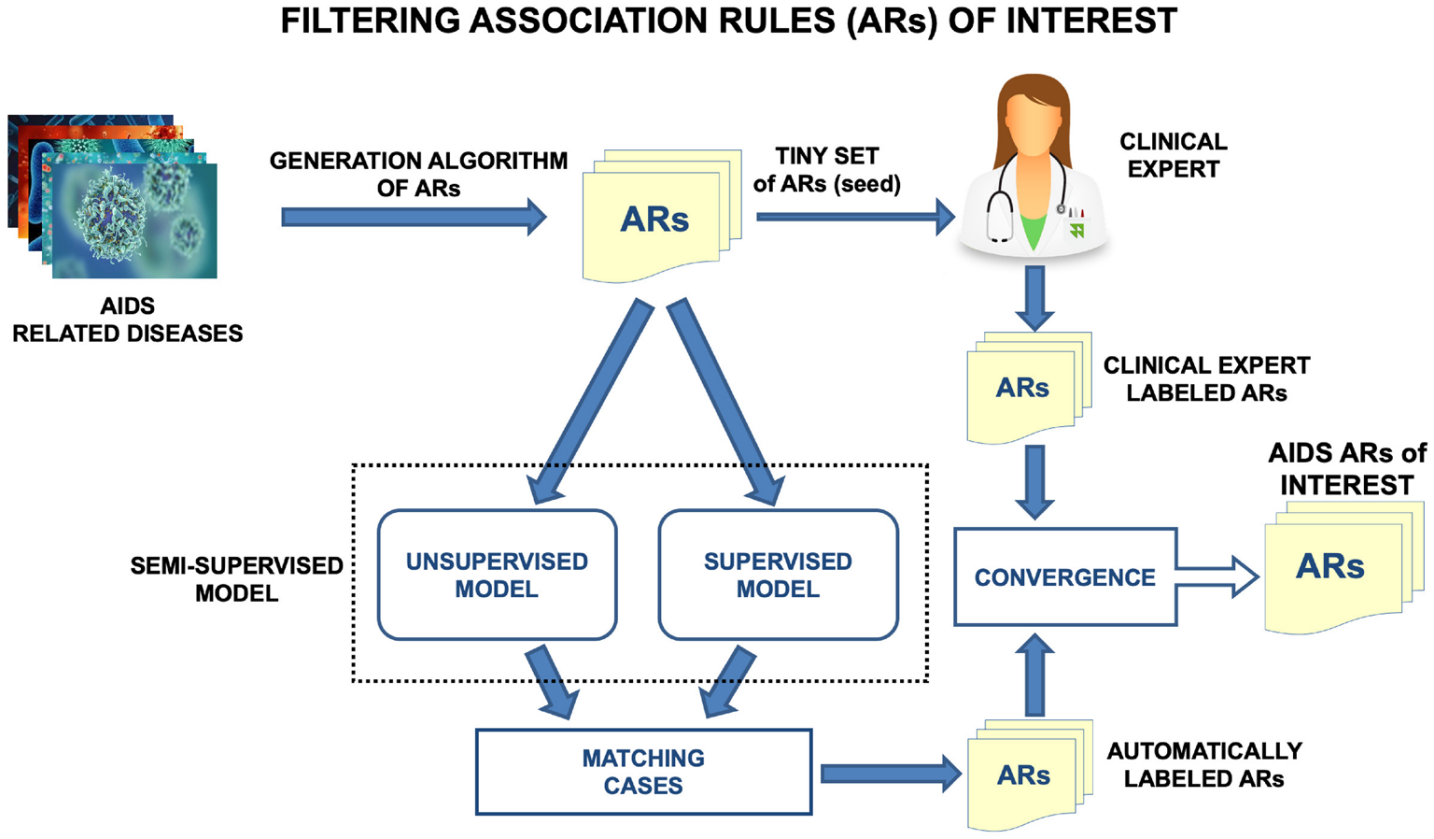
Scheme of EXTRAE, the semi-supervised learning model for the filtering of relevant ARs among HIV-related diseases.

Figure 1 shows a scheme of the applied semi-supervised system EXTRAE. From the data on the diseases suffered by each patient in the CoRIS database, all the ARs that satisfy minimum conditions of co-occurrence frequency (support and confidence) are generated. A medical expert evaluates a small set of these rules, which serves as a seed for the algorithm, as being relevant or not. That initial seed serves as a training set for a supervised algorithm. It also allows for certain adjustments of an unsupervised algorithm. When the predictions on the ARs of both algorithms match, they are considered sufficiently reliable to be added as reference data to the training set. The process is repeated until convergence is reached when no new ARs are added to the training set.

Our analysis has uncovered multiple relationships of interest between HIV-related diseases. Some of these relationships are well known to medical experts. Others are little known and, even though they could be confirmed in the literature, they were scattered and hence their compilation may represent a breakthrough in HIV research. Finally, other relationships, although plausible, are neither confirmed nor discarded in the literature and deserve to be studied in depth by medical professionals.

## Methods

In this section, we include details of the reference collection on HIV-related diseases. We also introduce the process followed to analyze and annotate the association rules to be used to train and evaluate the semi-supervised model. Finally, we present the semi-supervised model EXTRAE applied for ARs filtering.

### CORIS data

The Spanish HIV/AIDS research network (CoRIS) is an open, prospective, multicenter cohort of adult subjects with confirmed HIV infection, naïve to ART at study entry, recruited in 47 centers from 14 of 17 Autonomous Regions in Spain, from 2004 onwards. Data are organized and standardized following the HIV Cohorts Data Exchange Protocol (HICDEP) for data collection (details at https://hicdep.org/) and adhere to internal strict annual quality controls. The CoRIS database collects baseline and follow-up socio-demographic, immunological and clinical data. Patients are followed periodically according to routine clinical practice. The CoRIS cohort has been described in detail elsewhere^17^.

Each CoRIS participant provided his or her written informed consent prior to enrolling in this study. The CoRIS cohort was approved by the Research Ethic Committee of the Gregorio Marañón Hospital. All methods were carried out in accordance with relevant guidelines and regulations.

The HIV/AIDS Research Network Cohort was established in 2004 in conjunction with the HIV Biobank. These platforms are fundamental resources for improving knowledge about HIV. The coordination team is a multidisciplinary group: epidemiologists, statisticians, clinicians, pharmacists, microbiologist among others. The aim of CoRIS is to collect information on HIV-positive patients in order to study the epidemiological characteristics, the progression of the infection and its determinants, as well as the response to treatment and its influencing factors. CoRIS is organized around three structures:

- Coordination center: it organizes and coordinates data collection, supporting hospitals in these procedures and carries out data processing for statistical analysis.
- Clinical centers: hospitals and health centers where information and biological samples are collected.
- CoRIS Scientific Committee: it decides on the scientific development of the cohort. It also evaluates requests from research groups that need to use CoRIS data in their projects.
- Biobank: blood samples are collected (at baseline visit and annually thereafter) from all patients who give specific informed consent, from which aliquots of serum and cells are separated and stored in a centralized biobank.

For all patients enrolled in the cohort, updates of clinical data and biological parameters were requested at a periodicity of 6 ± 2 months. Follow-up was terminated upon death, change of follow-up center to one outside the cohort, or failure of the patient to appear for scheduled visits.

A database including all baseline and follow-up variables was created with the patient data and made available to all centers. After receiving the data, the coordinating center transfers them to a series of files with a common structure for all the centers. In order to connect all the information referring to the same patient and avoid duplication, a unique code combining the patient’s initials, date of birth and sex is used. The files are updated with the data sent by the hospitals every 12 months, both for new patients and for the follow-up of patients already included.

To guarantee the validity of the information and homogeneity between centers, several quality controls are carried out. All the information received at the data coordination center is subjected to a program that automatically detects inconsistencies, out-of-range data and duplicates. The cohort protocol was approved by the ethics committee of each participating hospital. When a patient is recruited, informed consent is requested. All information sent from the hospitals is anonymous.

In addition to clinical and epidemiological information, blood samples are collected from all patients who give specific informed consent, from which samples of serum and cells are separated and stored in a centralized BioBank for the entire cohort.

### Manual evaluation of association rules

The development of ML systems requires training data that allows the system to find the appropriate model and configuration to make predictions about new data. In our case, the objective is to have a ML system capable of discriminating whether an AR is relevant or not. For sake of simplicity, sometimes we will call them true (relevant) or false (otherwise). We therefore need a collection of ARs classified as true or false, which will allow us to perform the training. The first problem we faced was to establish the criteria for deciding whether an AR is relevant or not. After making a thorough study of the collection of initial rules extracted by the FP-Growth algorithm, we established the following set of criteria:

An AR R (X $\Rightarrow$ Y, X = ($x_1,\cdots ,x_n$)) is relevant (True) if

$$\begin{aligned} \exists i, i \in \{1,n\}, \text {such that } relation(x_i,Y) \text {is not trivial}. \end{aligned}$$

that is, if there is any non-trivial relationship between any of the antecedent and consequent diseases.

During the annotation process the following considerations have been taken into account:

- The association with the consequent can be either directly causal or inversely causal
  ($x_i \rightarrow Y$ and $Y \rightarrow x_i$ are considered equivalent):
  For example:
  acute myocardial infarction $\rightarrow$ high blood pressure: true
  but also:
  high blood pressure $\rightarrow$ acute myocardial infarction: true
  We assume that often the cause-effect relationship is not clearly established, so we simply consider the relationship between the two elements, without establishing which is the cause and which is the effect.
- If there are several antecedents and some of them are related to the consequent, even if they are not related to each other, the rule is considered true, that is
  for the AR R: X $\Rightarrow$ Y, X = ($x_1,\ldots ,x_n$)), relation($x_i, x_j$) are not taken into account.
  For example:
  *Arterial hypertension, Diabetes mellitus Dislipidemia* $\rightarrow$ *Acute myocardial infarction*
- If the association with the consequent is that it includes the antecedent or is a part of the antecedent (in a non-trivial way), the AR is true, that is
  if for the AR R: X $\Rightarrow$ Y, X = ($x_1,\ldots ,x_n$)) and $\exists i, \text {such that } i \in \{1,n\}, \text {such that } x_i$ is a disease that genalizes *Y* or vice versa, then the AR R is considered relevant:
  *lung neoplasm* $\rightarrow$ *Non-AIDS neoplasm*

In the first phase of this study, we considered that, if any of the elements included in the antecedent did not have an established relationship with the consequent, the rule was false. However, this led to eliminate many interesting rules in which an item could be included by chance, but which established a relevant relationship among the rest of the items. Therefore, we chose to classify as true those rules in which there were potentially relevant relations, even if they included some irrelevant items.

Once the general annotation guidelines were established, a HIV expert clinician (Dr. Otilia Bisbal) examined the rules, by gathering information to establish relationships between elements of the antecedent and the consequent based on their own experience in many cases, and also based on scientific literature in cases of less common relationships. Table 1 shows some data about the manually annotated ARs collection, Association Rules HIV/AIDS Dataset (ARAIDS). It is composed of 1000 rules of which, according to the adopted annotation criteria, 613 are relevant. The longest AR includes 4 antecedents and one consequent. The ARs in the collection involve 141 different health disorders, of which the one that occurs most frequently is *Non-defining neoplasm AIDS*, appearing 387 times.

**Table 1:** Some data on the Association Rules HIV/AIDS Dataset (ARAIDS), the manually validated collection of ARs.

Data	Description
Number of ARs	1000
ARs of interest	613
ARs without interest	387
Shortest AR	1 antecedent and 1 consequent
Longest AR	4 antecedents and 1 consequent
N. of health disorder involved	141
Most freq. health disorders in ARs	Non-defining neoplasm AIDS (387 times)

### Semisupervised method for filtering HIV/AIDS associated rules

This section presents the EXTRAE algorithm^16^, a semi-supervised algorithm which requires an extremely small amount of data to be trained. This system is made up of two modules or components: one that implements an unsupervised method and another that implements a supervised one.

The set of ARs are extracted from CoRIS data by means of the FP-Growth algorithm. This algorithm generates all the possible ARs with certain constraints related to the support and confidence conditions and to the form of the rules.

The support of an AR $X \Rightarrow Y$ is the fraction of transactions that include the set of items in the antecedent or consequent of the rule:

$$\begin{aligned} support(X \Rightarrow Y) = support(X \cup Y) = \frac{count(X \cup Y)}{N} \end{aligned}$$

where *N* is the number of transactions in the database, and count ($X \cup Y$) the number of transactions containing all items in *X* (antecedent) or Y (consequent).

On the other hand, the confidence of a rule is defined as the fraction of transactions in which itemsets X and Y appear:

$$\begin{aligned} conf(X \Rightarrow Y)= \frac{support(X \cup Y)}{support(X)} \end{aligned}$$

It can be interpreted as how often a transaction that contains the itemset X also contains itemset Y.

The parameters used for this algorithm are: Min. Support of 0.0001; Min. confidence of 0.6; Max. antecedent length unlimited; Max. consequent length of 1.

#### Unsupervised component of EXTRAE algorithm

This component applies Fisher’s exact test to obtain the p-value corresponding to the ARs. Specifically, the p-value is used to rank a set of ARs. We rank them in ascending order and establish a threshold. Then, the *n* rules above that threshold (lower value) are considered as true, and the rules below that threshold (higher value) as false.

In order to compute the p-value, the data set is split into two halves called exploratory (50%) and holdout (50%). Then, the FP-Growth algorithm is applied to extract the ARs in both sets.

These two sets of rules allow us to apply the Fisher test to obtain the p-values for the rules in the holdout set. This is done by building, for each rule R: $A \longrightarrow B$ in the holdout set, a contingency table with the following data for the rule collected in the exploratory dataset:

**Table:** 

	Rules with *B* ($n_2$)	Rules without *B* ($N - n_2$)
Rules with *A* ($n_1$)	Rules with *A* and *B* (*k*)	Rules with *A* (without *B*) ($n_1 - k$)
Rules without *A* ($$N - n_1)$$	Rules with *B* (without *A*) ($$n_2 - k$$)	Rules without *A* and *B* ($$N - n_1 - n_2 + k$$)

The p-value is computed as the hypergeometric distribution of the numbers contained in the cells of the table:

1 $$\begin{aligned} p(R) = \frac{\left( {\begin{array}{c}n_1\\ k\end{array}}\right) \left( {\begin{array}{c}N-n_1\\ n_2-k\end{array}}\right) }{\left( {\begin{array}{c}N\\ n_2\end{array}}\right) } \end{aligned}$$

where *N* is the number of rules in the exploratory set, *K* is the number of rules in this set containing *A* and *B*, $n_1$ is the number of rules containing *A*, $n_2$ is the number of rules containing *B*.

The value of the threshold is key to the performance of this unsupervised algorithm. Other studies using Fisher’s test for filtering the relevant ARs^14^ set the threshold following some heuristic such as taking the value which provides a certain number of relevant rules. However, in our case, it can be set with high accuracy using the data from the training set. The threshold is chosen so that the number of hits in the set of ARs of the training set is maximized. In this way, the unsupervised component becomes supervised to a certain degree. This is a great improvement for this method, which is later used to improve the results of the supervised component, resulting in a semi-supervised system that improves its two components.

#### Supervised component of EXTRAE algorithm

Thanks to the availability of manually annotated ARs, we can make use of classical ML systems to build a classifier that indicates whether a new rule is relevant. Specifically, we applied a Random Forest (default parameters provided by WEKA v3.8.2) with the following two groups of features:

- The first set of features have been extracted from the content of the association rules and the output of the FP-Growth algorithm:
  - Support.
  - Confidence.
  - Lift. The lift value is the quotient of the posterior and prior confidence of an association rule. That is, if “$\emptyset \rightarrow$ flu” has a confidence of 60% and “cough $\rightarrow$ flu” has a confidence of 72%, then the lift value (of the second rule) is 72/60 = 1.2.
  - Number of antecedents. The number of antecedents of an AR “A and B $\rightarrow$ C” is the number of elements of the set S ={ A, B }.
  - Number of consequents. The number of consequents of an AR “A and B $\rightarrow$ C” is the number of elements of the set S ={ C }.
- The second group of features attempts to capture medical information on diseases:
  - CDC. The Centers for Disease Control and Prevention (CDC) is the national public health agency of the United States and provides a list of diseases and conditions. This feature provides the normalised value of the association rule items that belong to this list.
  - DIS. In this feature, the items of the association rule that are diseases but do not appear in the CDC list are detected and the normalised value is provided.
  - ADD. The CoRIS dataset provides 34 adverse event types. These adverse events are a very serious type of disease like heart attck, lymphoma, cancer, etc.). This rule provides the normalised value of the presence of this type of adverse disease among the items of the association rule.
  - COD. The CoRIS dataset provides 123 types of cause of death. This feature identifies whether any item in the association rule belongs to this list and uniquely identifies it.
  - CIE. For each item of the association rule we have identified its ICD10 code. Due to the large number of possible labels of the complete code (more than 71K) we have only used the first character corresponding to the ”chapter”. Therefore we have created 24 features in which each rule indicates the number of items belonging to that ICD10 chapter.

#### Semi-supervised EXTRAE algorithm

**Figure 2:**
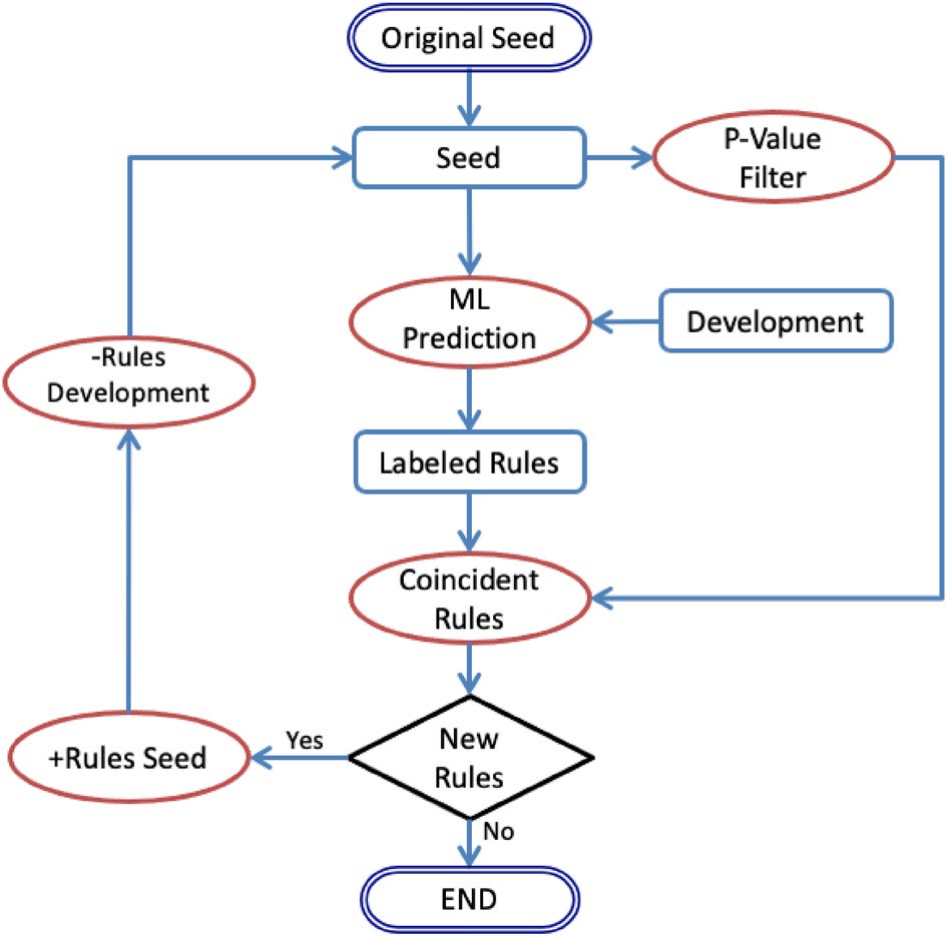
Flow diagram of incremental learning in EXTRAE semi-supervised algorithm for filtering relevant ARs. Rounded rectangles show the beginning and the end of the iterations, rectangles are the rule sets, ovals are processes, and the diamond represents a condition.

The semi-supervised combination of the components described above results in the semi-supervised EXTRAE system capable of classifying relevant ARs with high accuracy while requiring a minimal amount of training data. This system starts from a small seed dataset S of annotated rules. According to previous studies^16^, this initial seed can be set to around 10 rules. This seed set is used to train the supervised component leading to a ML model. This ML model is then used to predict the class (i.e. relevant or not) for each AR in the rest of the training set. The last computed seed dataset is also used to compute an accurate p-value threshold for the unsupervised component. After sorting S according to their p-value, we choose as threshold the p-value that maximizes the hits for the seed set. Next, the unsupervised component is applied to the development set to filter the relevant ARs. Afterwards, the results of both components, supervised and unsupervised, are applied to the development dataset for enlarging the seed set. Specifically, the ARs for which the predictions of both components match, i.e. both are true or both are false (coincident set of ARs), are added to the seed set and removed from the development set. The new seed set is used to train the supervised module again, as well as to adjust the threshold of the unsupervised component. This process is repeated until the coincident set of ARs is empty (i.e. the seed set cannot grow anymore). Figure 2 shows a scheme of the system.

## Results

In this section we present the experimental results obtained by the system on the ARAIDS dataset along with a parameter analysis.

For the evaluation of our system we have used a set of standard evaluation measures which focus on different aspects of the results. *F-measure* is a combination of the system precision and recall. *AUC ROC* estimates the area under the *ROC* curve for machine learning model comparison, where the *ROC* curve is a graph representing the performance of the system as its discrimination threshold for binary classification varies. We also use the *PR Curve (PRC)*, the result of drawing the graph between the precision and the recall. This graph shows from which recall we have a degradation of the precision and vice versa. The area under this curve (*AU-PRC*) provides a value to compare different systems.

We have carried out a fivefold cross-validation for evaluation. Since EXTRAE is a semi-supervised system, only a portion of the training rules are used for training (seed subset), depending on the different configurations analyzed.

Table 2 shows the results of the semi-supervised method based on Incremental Learning (EXTRAE Algorithm). In order to evaluate the results, the three evaluation measures that state-of-the-art systems typically employ (F-measure, AUR-ROC, and AU-PRC) are provided. As for other values shown in the table, seed size is the original size of the training set from which the set is automatically increased. Iterations show the number of times that new rules need to be added to the seed set, in order that a set is reached to which no new rule can be added. The *p-value* is calculated from the seed set. Results show the performance of the system after *n* iterations. From the results shown in this Table, the best seed size is 35. A *p-value* threshold of 1.12E−13 is calculated on this seed size and after 8 iterations an f-measure of 0.84 is obtained.

**Table 2:** Results of EXTRAE algorithm on ARAIDS dataset using different seed sizes, based on their F-measure, *AUC-ROC*, and *AU-PRC*.

ARAIDS dataset					
Seed size	Iterations	p-value	F-measure	AUC-ROC	AU-PRC
10	5	1.03E−12	0.62	0.69	0.70
15	4	1.03E−12	0.68	0.71	0.71
20	7	1.03E−12	0.72	0.77	0.77
25	5	1.12E−13	0.79	0.84	0.83
35	8	1.12E−13	**0.84**	**0.88**	**0.88**
50	9	1.12E−13	0.81	0.85	0.85
75	4	1.25E−13	0.80	0.84	0.83
100	6	1.25E−13	0.75	0.79	0.80
125	7	1.25E−13	0.76	0.81	0.81
150	5	1.25E−13	0.71	0.74	0.74

Iterations is the max. number of iterations reached and *p-value* is obtained automatically using the filter approach on the seed set. Best results appear in boldface.

Table 3 shows the partial results of the EXTRAE Algorithm in each iteration for the best configuration shown in Table 2. These results correspond to one of the 5 partitions used as part of the fivefold cross validation method. In the first iteration, 478 new rules are added and an F-measure of 0.72 is obtained. From the fifth iteration, the number of matching rules is greatly reduced and, in this way, the performance increases slowly until it reaches an F-measure of 0.84. In only eight iterations it increases its performance by 75%, which proves the high quality of the added rules. If we look at the AUC-ROC and AU-PRC, the final results are even higher than the F-measure. This shows that the system performance is robust when using different evaluation criteria.

**Table 3:** Evolution of learning from a seed set with 35 rules from one of the 5 partitions used as part of the fivefold cross validation, based on their F-Measure, *AUC-ROC*, and *AU-PRC*.

Iteration	Coincident rules	F-measure	AUC-ROC	AU-PRC
0	–	0.48	0.53	0.54
1	478	0.72	0.79	0.78
2	156	0.79	0.83	0.84
3	41	0.81	0.84	0.84
4	18	0.81	0.85	0.85
5	9	0.82	0.85	0.85
6	6	0.82	0.86	0.86
7	5	0.83	0.87	**0.88**
8	2	**0.84**	**0.88**	**0.88**

Coincident rules are those from the development set that have the same prediction and label based on the p-value filter. Best results appear in boldface.

Table 4 shows the results of the EXTRAE system compared to the two supervised and unsupervised systems of which EXTRAE is composed. For the supervised system, results are provided when using the same number of seeds (35) as used by EXTRAE, and also for the maximum number of association rules (80%) of the training set. Note that the test set is 20% of the dataset. Finally, the results of the unsupervised system are shown taking into account the best performing p-value. The unsupervised system performs significantly worse than EXTRAE, which shows the complexity of the problem. In the case of the supervised system trained with 35 rules, it was clear that the amount of training data was too low for a supervised system, but we wanted to reflect the power of EXTRAE when using the same number of rules.

Finally, EXTRAE obtains better results than the supervised system trained with 800 rules by a slight difference. This comparison is most interesting because, despite initially training with a seed of 35 rules, EXTRAE manages to select the most discriminating rules from the training set to end up with a higher quality rule set than the supervised system and, thus, improve its performance.

**Table 4:** Results of the best pereformance for the EXTRAE algorithm, and both supervised and unsupervised systems on ARAIDS dataset using the best configuration of EXTRAE, based on their F-measure, *AUC-ROC*, and *AU-PRC*.

ARAIDS dataset						
System	Seed/training size	Iterations	p-value	F-measure	AUC-ROC	AU-PRC
EXTRAE	35 rules	8	1.12E−13	**0.84**	**0.88**	**0.88**
Supervised	35 rules	**–**	**–**	0.52	0.56	0.55
Supervised	800 rules	**–**	**–**	0.81	0.86	0.85
Unsupervised	**–**	**–**	5.32E−14	0.68	**–**	**–**

Iterations is the max. number of iterations reached and *p-value* is obtained automatically using the filter approach on the seed set for EXTRAE. Best results appear in boldface.

## Discussion

In this study, we have proven that it is possible to perform filtering of relevant ARs with high accuracy, using a semi-supervised system capable of operating on an extremely small amount of training data. Specifically, in this case, the optimal results have been reached with only 35 annotated ARs. The results for the data considered here are even better than those obtained by the EXTRAE algorithm applied to other data in a previous study^16^. In the latter, the semi-supervised algorithm applied to primary care data obtained an F-measure of 0.75, AUC-ROC of 0.80 and AU-PRC of 0.81 respectively.

We have demonstrated that it is advantageous to perform association rule filtering with a semi-supervised system, and that the results of such a system are able to outperform both unsupervised and supervised systems when using a reduced amount of training data.

Association rules are a fairly simple artifact in their form, unlike, for example, texts. Therefore, a small number of parameters is enough to characterize their form, as well as aspects related to the frequency of the diseases involved and their combinations (captured by the features of support and confidence).

The medical aspects of the system are to a great extent captured by the unsupervised part. In this part, the system includes the most statistically significant ARs according to the diseases that make up each rule. The combination of both parts and their joint evolution leads the system to select the most relevant ARs.

The prediction of relevant ARs provided by the proposed model and its validation by a HIV medical expert has provided an interesting collection of HIV-related disease relationships. We describe below a number of relationships that have appeared during the validation process.

The relationship of depression with cardiovascular disease, diabetes and cancer is described in the literature and is probably due to immune factors, toxic habits, drugs, etc. and has, therefore, been considered to be true. Similarly, the relationship between psychosis with cardiovascular risk and diabetes has also been described for the same reasons and has also been considered to be true^18–21^.

The relationship between acute myocardial infarction (AMI) and dementia (especially when it is of vascular origin) has also been described in the literature, so it has been considered true^22,23^.

Likewise, the relationship between diabetes and dementia is also reported in the literature, so it has been considered true^24^.

A doubtful case is the relationship between fracture and neoplasm: in extended neoplasms when bone metastases occur there may be pathological fractures, but this is not frequent, and it is not the type of fracture referred to in the CoRIS database, so it has been considered false.

The relationship between Kaposi’s sarcoma and bronchial neoplasm has been considered true since Kaposi’s sarcoma is a neoplasm that can affect the lung.

The relationship between cachectic syndrome (cachectic sd) and neoplasms has been considered false, because even though the latter can produce a cachectic sd, in the CoRIS database it is specified that it refers to the former due to HIV or wasting syndrome in particular.

Regarding the term “secondary malignant neoplasm of other specified sites”, if it appears in an AR whose consequent is lung or bladder cancer or head and neck cancer, the AR has been considered true because it can be a metastasis of these neoplasms. However, it has been considered false if the consequent of AR is recurrent bacterial pneumonia, because although the latter increases the risk of lung neoplasm, it does not increase the risk of any neoplasm.

The relationship between lactic acidosis and diabetes and cancer has been described^25^ so it has been considered true.

Appendix A includes a series of relationships considered to be true since they have been confirmed in the literature, together with some references to them. The appendix also gathers relationships that have been considered false either because they lack consistent support in the literature or because they have been ruled out by the literature.

## Supplementary Information

Supplementary Information.

## Data Availability

The datasets supporting the conclusions of this article are included within the article and its tables, as well as in the supplementary material. The original HIV data from the CoRIS Cohort can be requested to this organization under the corresponding agreement.
