# Supplementary material for: Discovering HIV related information by means of association rules and machine learning
Source: Sci Rep. 2022 Oct 28;12:18208. doi: 10.1038/s41598-022-22695-y (PMC9616424; doi:10.1038/s41598-022-22695-y)
Supplement: Supplementary file 1 — Supplementary Information. [file 41598_2022_22695_MOESM1_ESM.pdf]

Discovering HIV related information by means of association rules and machine learning

Lourdes Araujo[1,4,*], Juan Martinez-Romo[1,4], Otilia Bisbal[2,4], Ricardo Sanchez-de-Madariaga[3,4], and The Cohort of the National AIDS Network (CoRIS)[+]

[1]Languages and Information Systems Dpt., ETS Ingeniería Informática (UNED), Juan del Rosal 16, 28040 Madrid, Spain
[2]Hospital Universitario 12 de Octubre. Instituto de Investigación I+12
[3]Telemedicine and e-Health Research Unit, Instituto de Salud Carlos III, Monforte de Lemos 5, 28029 Madrid, Spain
[4]Instituto Mixto UNED-ISCIII IMIENS, 28029 Madrid, Spain
[+]See Annex B with the list of all participating centers and researchers.
[*]lurdes@lsi.uned.es

SUPPLEMENTARY MATERIAL

1 Additional relationships confirmed or discarded

Other relationships, presented in the Table 1 have been considered true during the annotation of the rules as confirmed by the literature. In contrast, the relationships appearing in Table 2 have been considered false, either because there is no consistent literature or because there is literature that rules them out.

2 The Cohort of the National AIDS Network CORIS

Executive committee: Santiago Moreno, Inma Jarrín, David Dalmau, M Luisa Navarro, M Isabel González, Federico Garcia, Eva Poveda, Jose Antonio Iribarren, Félix Gutiérrez, Rafael Rubio, Francesc Vidal, Juan Berenguer, Juan González, M Ángeles Muñoz-Fernández.

Fieldwork data management and analysis: Inmaculada Jarrín, Cristina Moreno, Marta Rava, Rebeca Izquierdo.

BioBanK: HIV Hospital General Universitario Gregorio Marañón: M Ángeles Muñoz-Fernández, Elba Mauleón.

Participants:

Joaquín Portilla[1], Irene Portilla[1], Esperanza Merino[1], Gema García[1], Iván Agea[1], José Sánchez-Payá[1], Juan Carlos Rodríguez[1], Livia Giner[1], Sergio Reus[1], Vicente Boix[1], Diego Torrus[1], Verónica Pérez[1], Julia Portilla[1], Juan Luís Gómez[2], Jehovana Hernández[2], Ana López Lirola[2], Dácil García[2], Felicitas Díaz-Flores[2], M Mar Alonso[2], Ricardo Pelazas[2], M Remedios Alemán[2]. Víctor Asensi[3], María Eugenia Rivas Carmenado[3], Tomás Suarez-Zarracina[3], Federico Pulido[4], Rafael Rubio[4], Otilia Bisbal[4], M Asunción Hernando[4], David Rial[4], María de Lagarde[4], Octavio Arce[4], Adriana Pinto[4], Laura Bermejo[4], Mireia Santacreu[4], Roser Navarro[4], Candela Gonzalez[4], Jose Antonio Iribarren[5], M José Aramburu[5], Xabier Camino[5], Miguel Ángel von Wichmann[5], Miguel Ángel Goenaga[5], M Jesús Bustinduy[5], Harkaitz Azkune[5], Maialen Ibarguren[5], Xabier Kortajarena[5], Ignacio Álvarez-Rodriguez[5], Leire Gil[5], Lourdes Martínez[5], Félix Gutiérrez[6], Catalina Robledano[6], Mar Masiá[6], Sergio Padilla[6], Araceli Adsuar[6], Rafael Pascual[6], Marta Fernández[6], Antonio Galiana[6], José Alberto García[6], Xavier Barber[6], Vanessa Agullo[6], Javier Garcia Abellán[6], Reyes Pascual[6], Guillermo Telenti[6], Lucia Guillén[6], Ángela Botella[6], Roberto Muga[7], Arantza Sanvisens[7], Daniel Fuster[7], Juan Berenguer[8], Isabel Gutierrez[8], Juan Carlos López[8], Margarita Ramírez[8], Belén Padilla[8], Paloma Gijón[8], Teresa Aldamiz-Echevarría[8], Francisco Tejerina[8], Cristina Diez[8], Leire Pérez[8], Chiara Fanciulli[8], Saray Corral[8] Francesc Vidal[9], Anna Martí[9], Joaquín Peraire[9], Consuelo Viladés[9], Montserrat Vargas[9], Montserrat Olona[9], Anna Rull[9], Verónica Alba[9], Elena Yeregui[9], Jenifer Masip[9], Graciano García-Pardo[9], Frederic Gómez

[1]Hospital General Universitario de Alicante (Alicante), Spain
[2]Hospital Universitario de Canarias (San Cristóbal de la Laguna), Spain
[3]Hospital Universitario Central de Asturias (Oviedo), Spain
[4]Hospital Universitario 12 de Octubre (Madrid), Spain
[5]Servicio de Enfermedades Infecciosas. Hospital Universitario Donostia. Instituto de Investigación BioDonostia (Donostia-San Sebastián), Spain
[6]Hospital General Universitario De Elche (Elche), Spain
[7]Hospital Universitari Germans Trias i Pujol (Can Ruti) (Badalona), Spain
[8]Hospital General Universitario Gregorio Marañón (Madrid), Spain
[9]Hospital Universitari de Tarragona Joan XXIII (Tarragona), Spain

Disease 1	Disease 2	Reference
neoplasia	depression	1
Psychosis	Diabetes mellitus, hypertension arterial, acute myocardial infarction	2
Psychosis	fracture	3
Acute renal failure	depression	4
DM(Diabetes mellitus)	cancer	5
acute myocardial infarction	cancer	6
cerebrovascular disease	cancer	7
suicide	cancer	8
Coronary heart disease	cancer	9
Peripheral arterial disease	cancer	10
hipertensión arterial	cancer	11
Lactic acid	cancer	12
Tuberculosis	cancer	13
Mycobacterium avium intracellulare	cancer	14
Pneumonia	cancer	15
Prostate neoplasia	acute myocardial infarction	16
Dementia	cancer	17
Diabetes Mellitus	HIV encephalopathy	18
Diabetes Mellitus	Kaposi's sarcoma	19
Diabetes Mellitus	depression	20
Recurrent pneumonia	myocardial infarction	21
Fracture	acute myocardial infarction	22
Tuberculosis	acute myocardial infarction	23
Tuberculosis	cytomegalovirus infection	24
Peripheral arterial disease	coronary artery disease; cirrhosis	25
Fracture	depression	26
Leishmania	depression	27
Acute renal failure	dementia	28
Nephrolithiasis	Diabetes Mellitus; hipertensión arterial	29
Nephrolithiasis	acute myocardial infarction	30
Nephrolithiasis	Cerebrovascular disease	31
Acute renal failure	leishmaniasis	32
Acute renal failure	Fracture	33
Mycobacterium avium intracellulare	Acute renal failure	34
Kaposi	Acute renal failure	35
Fracture	diabetes	36

Table 1. Some additional disease relationships confirmed in the literature

.

Disease 1	Disease 2	Comment
Renal lithiasis	cancer, Hemangioma and non-AIDS neoplasm, Hemangioma and benign vascular tumor	
Primary pulmonary hypertension	cancer	
Lung cancer	cirrhosis	
Primary pulmonary hypertension	cirrhosis	the relationship is with pulmonary hypertension in general, not primary
Leishmania	acute myocardial infarction	
Mycobacterium Avium-intracellulare	acute myocardial infarction	
HIV encephalopathy	acute myocardial infarction	
Encephalopathy	tuberculosis	
Acute renal failure	Psychosis	
Pneumocystys	acute myocardial infarction	
HIV encephalopathy	cancer	However, there is a relationship of encephalopathy related to dementia and of dementia with cancer
acute myocardial infarction	Non-Hodking Lymphoma	
extrapulmonary cryptococcosis	acute respiratory infection	
Leishmaniasis	Diabetes mellitus	
Kaposi's disease	acute myocardial infarction	
CHF (chronic heart failure)	Pneumocystis	[37]
Lactic acidosis	fracture	
Lactic acidosis	Pneumocystis	[38] However there is a relation between pneumocystis treatment (Soltrim) and Lactic acidosis
Lactic acidosis	cryptococcosis	
Pneumocystis	cancer	

Table 2. Some excluded disease relationships

.

Bertomeu[9], Sonia Espineira[9], Marta Montero[10], Sandra Cuéllar[10], Marino Blanes[10], María Tasias[10], Eva Calabuig[10], Miguel Salavert[10], Juan Fernández[10], Inmaculada Segarra[10], Juan González-García[11], Ana Delgado-Hierro[11], José Ramón Arribas[11], Victor Arribas[11], Jose Ignacio Bernardino[11], Carmen Busca[11], Joanna Cano[11], Julen Cardiñanos[11], Juan Miguel Castro[11], Luis Escosa[11], Iker Falces[11], Pedro Herranz[11], Victor Hontañón[11], Milagros García[11], Alicia González-Baeza[11], Mª Luz Martín-Carbonero[11], Mario Mayoral[11], Mª Jose Mellado[11], Rafael Micán[11], Rosa de Miguel[11], Rocío Montejano[11], Mª Luisa Montes[11], Victoria Moreno[11], Luis Ramos[11], Berta Rodés[11], Talía Sainz[11], Elena Sendagorta[11], Eulalia Valencia[11], Jose Ramón Blanco[12], Laura Pérez-Martínez[12], José Antonio Oteo[12], Valvanera Ibarra[12], Luis Metola[12], Mercedes Sanz[12], Piedad Arazo[13], Gloria Sampériz[13], David Dalmau[14], Marina Martinez[14], Angels Jaén[14], Montse Sanmartí[14], Mireia Cairó[14], Javier Martinez-Lacasa[14], Pablo Velli[14], Roser Font[14], Mariona Xercavins[14], Noemí Alonso[14], Francesco Aiello[14], María Rivero[15], Beatriz Piérola[15], Maider Goikoetxea[15], María Gracia[15], Carlos Ibero[15], Estela Moreno[15], Jesús Repáraz[15], Gemma Navarro[16], Manel Cervantes Garcia[16], Sonia Calzado Isbert[16], Marta Navarro Vilasaro[16], Belen Lopez Garcia[16], Ignacio de los Santos[17], Alejandro de los Santos[17], Jesús Sanz[17], Lucio García-Fraile[17], Enrique Martín[17], Ildefonso Sánchez-Cerrillo[17], Marta Calvet[17], Ana Barrios[17], Azucena Bautista[17], Carmen Sáez[17], Marianela Ciudad[17], Ángela Gutiérrez[17], Santiago Moreno[18], Santos del Campo[18], José Luis Casado[18], Fernando Dronda[18], Ana Moreno[18], M Jesús Pérez[18], Sergio Serrano[18], Mª Jesús Vivancos[18], Javier Martínez-Sanz[18], Alejandro Vallejo[18], Matilde Sanchez[18], Jose Antonio Pérez-Molina[18], José Manuel Hermida[18], Enrique Bernal[19], Antonia Alcaraz[19], Joaquín Bravo[19], Ángeles Muñoz[19], Cristina Tomás[19], Mónica Martínez[19], M Carmen Villalba[19], Federico García[20], Clara Martínez[20], José Hernández[20], Leopoldo Muñoz Medina[20], Marta Álvarez[20], Natalia Chueca[20], David Vinuesa[20], Adolfo de Salazar[20], Ana Fuentes[20], Emilio Guirao[20], Laura Viñuela[20], Andrés Ruiz-Sancho[20], Francisco Anguita[20], Jorge Del Romero[21], Montserrat Raposo[21], Carmen Rodríguez[21], Teresa Puerta[21], Juan Carlos Carrió[21], Mar Vera[21], Juan Ballesteros[21], Oskar Ayerdi[21], Begoña Baza[21], Eva Orviz[21], Antonio Antela[22], Elena Losada[22], Melchor Riera[23], María Peñaranda[23], M Angels Ribas[23], Antoni A. Campins[23], Mercedes Garcia-Gazalla[23], Francisco J Fanjul[23], Javier Murillas[23], Francisco Homar[23], Helem H Vilchez[23], Luisa Martin[23], Antoni Payeras[23], Jesús Santos[24], María López[24], Crisitina Gómez[24], Isabel Viciana[24], Rosario Palacios[24], Luis Fernando López-Cortés[25], Nuria Espinosa[25], Cristina Roca[25], Silvia Llaves[25], Juan Manuel Tiraboschi[26], Arkaitz Imaz[26], Ana Karina Silva[26], María Saumoy[26], Sofía Catalina Scévola[26], Adrián Curran[27], Vicenç Falcó[27], Jordi Navarro[27], Joaquin Burgos[27], Paula Suanzes[27], Jorge García[27], Vicente Descalzo[27], Patricia Álvarez[27], Bibiana Planas[27], Marta Sanchiz[27], Lucía Rodríguez[27], Julián Olalla[28], M José Sánchez[28], Javier Pérez[28], Alfonso del Arco[28], Javier de la Torre[28], José Luis Prada[28], Onofre Juan Martínez[29], Lorena Martinez[29], Francisco Jesús Vera[29], Josefina García[29], Begoña Alcaraz[29], Antonio Jesús Sánchez Guirao[29], Alvaro Mena[30], Angeles Castro[30], Berta Pernas[30], Pilar Vázquez[30], Soledad López[30], Sofía Ibarra[31], Guillermo García[31], Josu Mirena[31], Oscar Luis Ferrero[31], Josefina López[31], M Mar Cámara[31], Mireia de la Peña[31], Miriam Lopez[31], Iñigo Lopez[31], Itxaso Lombide[31], Victor Polo[31], Joana de Miguel[31], Carlos Galera[32], Marian Fernández[32], Helena Albendin[32], Antonia Castillo[32], Asunción Iborra[32], Antonio Moreno[32], M Angustias Merlos[32], Asunción Vidal[32], Concha Amador[33], Francisco Pasquau[33], Concepcion Gil[33], Jose Tomás Algado[33], Inés Suarez-García[34], Eduardo Malmierca[34], Patricia González-Ruano[34], M Pilar Ruiz[34], José Francisco Pascual[34], Elena

[10]Hospital Universitario y Politécnico de La Fe (Valencia), Spain
[11]Hospital Universitario La Paz/IdiPAZ (Madrid), Spain
[12]Hospital San Pedro Centro de Investigación Biomédica de La Rioja (CIBIR) (Logroño), Spain
[13]Hospital Universitario Miguel Servet (Zaragoza), Spain
[14]Hospital Universitari Mutua Terrassa (Terrassa), Spain
[15]Complejo Hospitalario de Navarra (Pamplona), Sapin
[16]Parc Taulí Hospital Universitari (Sabadell), Spain
[17]Hospital Universitario de La Princesa (Madrid), Spain
[18]Hospital Universitario Ramón y Cajal (Madrid), Spain
[19]Hospital General Universitario Reina Sofía (Murcia), Spain
[20]Hospital Nuevo San Cecilio (Granada), Spain
[21]Centro Sanitario Sandoval (Madrid), Spain
[22]Hospital Clínico Universitario de Santiago (Santiago de Compostela), Spain
[23]Hospital Universitario Son Espases (Palma de Mallorca), Spain
[24]Hospital Universitario Virgen de la Victoria (Málaga), Spain
[25]Hospital Universitario Virgen del Rocío (Sevilla), Spain
[26]Hospital Universitario de Bellvitge (Hospitalet de Llobregat), Spain
[27]Hospital Universitario Valle de Hebrón (Barcelona), Spain
[28]Hospital Costa del Sol (Marbella), Spain
[29]Hospital General Universitario Santa Lucía (Cartagena), Spain
[30]Complejo Hospitalario Universitario a Coruña (Chuac) (A Coruña), Spain
[31]Hospital Universitario Basurto (Bilbao), Spain
[32]Hospital Universitario Virgen de la Arrixaca (El Palmar), Spain
[33]Hospital de la Marina Baixa (La Vila Joiosa), Spain
[34]Hospital Universitario Infanta Sofía (San Sebastián de los Reyes), Spain

Sáez[34], Luz Balsalobre[34], M Villa López[35], Mohamed Omar[35], Carmen Herrero[35], M Amparo Gómez[35], Miguel Alberto de Zarraga[36], Desiré Pérez[36], Vicente Estrada[37], Nieves Sanz[37], Noemí Cabello[37], Jorge Vergas García[37], Maria Jose Núñez[37], Iñigo Sagastagoitia[37], Miguel Górgolas[38], Alfonso Cabello[38], Beatriz Álvarez[38], Laura Prieto[38], Irene Carrillo[38], José Sanz[39], Alberto Arranz[39], Cristina Hernández[39], María Novella[39], M José Galindo[40], Ana Ferrer[40], Antonio Rivero Román[41], Inma Ruíz[41], Antonio Rivero Juárez[41], Pedro López[41], Isabel Machuca[41], Mario Frias[41], Ángela Camacho[41], Ignacio Pérez[41], Diana Corona[41], Ignacio Pérez[41], Diana Corona[41], Miguel Cervero[42], Rafael Torres[42], Juan Antonio Pineda[43], Pilar Rincón[43], Juan Macías[43], Luis Miguel Real[43], Anais Corma[43], Marta Fernández[43], Alejandro Gonzalez-Serna[43], Eva Poveda[44], Alexandre Pérez[44], Luis Morano[44], Celia Miralles[44], Antonio Ocampo[44], Guillermo Pousada[44], Lucía Patiño[44], Carlos Dueñas[45], Sara Gutiérrez[45], Elena Tapia[45], Cristina Novoa[45], Xjoylin Egües[45], Pablo Telleria[45].

References

1. Krebber, A. *et al.* Prevalence of depression in cancer patients: a meta-analysis of diagnostic interviews and self-report instruments. *Psycho-Oncology* **23**, 121–130 (2014).
2. De Hert, M. *et al.* Cardiovascular disease and diabetes in people with severe mental illness position statement from the european psychiatric association (epa), supported by the european association for the study of diabetes (easd) and the european society of cardiology (esc). *Eur. psychiatry* **24**, 412–424 (2009).
3. Forns, J. *et al.* Increased risk of falls and fractures in patients with psychosis and parkinson disease. *PloS one* **16**, e0246121 (2021).
4. Balogun, R. A. *et al.* Major depression and long-term outcomes of acute kidney injury. *Nephron* **135**, 23–30 (2017).
5. Gallagher, E. J. & LeRoith, D. Obesity and diabetes: the increased risk of cancer and cancer-related mortality. *Physiol. reviews* **95**, 727–748 (2015).
6. Rinde, L. B. *et al.* Myocardial infarction and future risk of cancer in the general population—the tromsø study. *Eur. journal epidemiology* **32**, 193–201 (2017).
7. Adams, H. P. Cancer and cerebrovascular disease. *Curr. Neurol. Neurosci. Reports* **19**, 1–10 (2019).
8. Stiefel, F., Volkenandt, M. & Breitbart, W. Suicide and cancer. *Schweizerische Medizinische Wochenschrift* **119**, 891–895 (1989).
9. Das, D., Asher, A. & Ghosh, A. K. Cancer and coronary artery disease: common associations, diagnosis and management challenges. *Curr. Treat. Options Oncol.* **20**, 1–12 (2019).
10. Bintein, F. *et al.* Patients with atherosclerotic peripheral arterial disease have a high risk of lung cancer: Systematic review and meta-analysis of literature. *JMV-Journal de Médecine Vasc.* **46**, 53–65 (2021).
11. Milan, A. *et al.* Arterial hypertension and cancer. *Int. journal cancer* **134**, 2269–2277 (2014).
12. Dhup, S., Kumar Dadhich, R., Ettore Porporato, P. & Sonveaux, P. Multiple biological activities of lactic acid in cancer: influences on tumor growth, angiogenesis and metastasis. *Curr. pharmaceutical design* **18**, 1319–1330 (2012).
13. Falagas, M., Kouranos, V., Athanassa, Z. & Kopterides, P. Tuberculosis and malignancy. *QJM: An Int. J. Medicine* **103**, 461–487 (2010).
14. Pierce, E. S. Baseballs, tennis balls, livestock farm manure, the idh1 mutation, endothelial cell proliferation and hypoxic pseudopalisading (granulomatous) necrosis: Mycobacterium avium subspecies paratuberculosis and the epidemiology, cellular metabolism and histolog. *Open Vet. J.* **9**, 5–12 (2019).
15. Wong, J. L. & Evans, S. E. Bacterial pneumonia in patients with cancer: novel risk factors and management. *Clin. chest medicine* **38**, 263–277 (2017).

[35]Hospital Universitario de Jaén (Jaén), Spain
[36]Hospital Universitario San Agustín (Avilés), Spain
[37]Hospital Clínico San Carlos (Madrid), Spain
[38]Hospital Universitario Fundación Jiménez Díaz (Madrid), Spain
[39]Hospital Universitario Príncipe de Asturias (Alcalá de Henares), Spain
[40]Hospital Clínico Universitario de Valencia (Valencia), Spain
[41]Hospital Reina Sofía (Córdoba), Spain
[42]Hospital Universitario Severo Ochoa (Leganés), Spain
[43]Nuestra Señora de Valme (Sevilla), Spain
[44]Hospital Álvaro Cunqueiro (Vigo), Spain
[45]Hospital Clínico Universitario de Valladolid (Valladolid), Spain

16. Hu, J.-R. *et al.* Cardiovascular effects of androgen deprivation therapy in prostate cancer: contemporary meta-analyses. *Arter. thrombosis, vascular biology* **40**, e55–e64 (2020).

17. van der Willik, K. D., Schagen, S. B. & Ikram, M. A. Cancer and dementia: Two sides of the same coin? *Eur. journal clinical investigation* **48**, e13019 (2018).

18. McCutchan, J. *et al.* Role of obesity, metabolic variables, and diabetes in hiv-associated neurocognitive disorder. *Neurology* **78**, 485–492 (2012).

19. Chang, P.-J. *et al.* Diabetes and risk of kaposi's sarcoma: effects of high glucose on reactivation and infection of kaposi's sarcoma-associated herpesvirus. *Oncotarget* **8**, 80595 (2017).

20. Roy, T. & Lloyd, C. E. Epidemiology of depression and diabetes: a systematic review. *J. affective disorders* **142**, S8–S21 (2012).

21. Cangemi, R. *et al.* Corticosteroid use and incident myocardial infarction in adults hospitalized for community-acquired pneumonia. *Annals Am. Thorac. Soc.* **16**, 91–98 (2019).

22. Pedersen, A. B., Ehrenstein, V., Szépligeti, S. K. & Sørensen, H. T. Hip fracture, comorbidity, and the risk of myocardial infarction and stroke: a danish nationwide cohort study, 1995–2015. *J. Bone Miner. Res.* **32**, 2339–2346 (2017).

23. Huaman, M. A. *et al.* The relationship between latent tuberculosis infection and acute myocardial infarction. *Clin. Infect. Dis.* **66**, 886–892 (2018).

24. Cobelens, F., Nagelkerke, N. & Fletcher, H. The convergent epidemiology of tuberculosis and human cytomegalovirus infection. *F1000Research* **7** (2018).

25. Lin, S.-Y. *et al.* Risk of acute coronary syndrome and peripheral arterial disease in chronic liver disease and cirrhosis: a nationwide population-based study. *Atherosclerosis* **270**, 154–159 (2018).

26. Heidari, M. E. *et al.* Prevalence of depression in older people with hip fracture: a systematic review and meta-analysis. *Int. J. Orthop. Trauma Nurs.* **40**, 100813 (2021).

27. Pires, M., Wright, B., Kaye, P. M., da Conceição, V. & Churchill, R. C. The impact of leishmaniasis on mental health and psychosocial well-being: a systematic review. *PLoS One* **14**, e0223313 (2019).

28. Hussain, S. *et al.* Association of acute kidney injury with the risk of dementia: A meta-analysis. *J. clinical medicine* **10**, 4390 (2021).

29. Lin, B.-B. *et al.* Associations between nephrolithiasis and diabetes mellitus, hypertension and gallstones: A meta-analysis of cohort studies. *Nephrology* **25**, 691–699 (2020).

30. Karami, H. *et al.* A short review on the relationships between nephrolithiasis and myocardial infarction. *Galen Med. J.* **8**, e1289 (2019).

31. Peng, J.-P. & Zheng, H. Kidney stones may increase the risk of coronary heart disease and stroke: A prisma-compliant meta-analysis. *Medicine* **96** (2017).

32. Oliveira, M. J. *et al.* Risk factors for acute kidney injury in visceral leishmaniasis (kala-azar). *The Am. journal tropical medicine hygiene* **82**, 449 (2010).

33. Berar Yanay N, B. Y., Abu Arisha M. Acute kidney injury in patients undergoing hip fracture surgery. *The Isr. Med. Assoc. Journal: IMAJ* **23**, 815–818 (2021).

34. Rawla, M., Kozak, A., Hadley, S. & LeCates, W. Mycobacterium avium-intracellulare-associated acute interstitial nephritis: a rare cause of renal allograft dysfunction. *Transpl. Infect. Dis.* **11**, 529–533 (2009).

35. Story, M. T. *et al.* Infiltrating kaposi sarcoma presenting as acute kidney injury: An unexpected consequence of deliberate hepatitis c–positive organ transplantation. *Transpl. Infect. Dis.* **23**, e13481 (2021).

36. Kurra, S., Fink, D. A. & Siris, E. S. Osteoporosis-associated fracture and diabetes. *Endocrinol. Metab. Clin.* **43**, 233–243 (2014).

37. Merino-Casallo, I. *et al.* Pneumocystis jirovecii in spanish patients with heart failure. *Front. Public Heal.* 289 (2019).

38. Bulathsinghala, M., Keefer, K. & Van de Louw, A. Trimethoprim/sulfamethoxazole-induced severe lactic acidosis: a case report and review of the literature. *Medicine* **95** (2016).
